# A new species in the *Tetramorium solidum*-group (Hymenoptera, Formicidae, Myrmicinae) from the Richtersveld National Park, South Africa, with an assessment of threats and conservation status

**DOI:** 10.3897/zookeys.965.52735

**Published:** 2020-09-03

**Authors:** Peter G. Hawkes

**Affiliations:** 1 AfriBugs CC, 341 27th Avenue, Villieria, Pretoria, Gauteng Province, 0186, South Africa Afribugs Pretoria South Africa; 2 Department of Zoology, University of Venda, Thohoyandou, Limpopo Province, South Africa University of Venda Thohoyandou South Africa

**Keywords:** Afrotropical, climate change, conservation, livestock, mining, taxonomy

## Abstract

Eight specimens of an undescribed species of *Tetramorium* in the *T. solidum*-group were collected by pitfall trapping during an environmental impact assessment survey of the Oena Diamond Mine in the Richtersveld National Park, South Africa. The new species is most closely related to *T. grandinode* Santschi, 1913 but can be distinguished from this species by the distinctly different patterns of costulate sculpture on the mesosomal dorsum and petiole node, as well as the different forms of the petiole and postpetiole nodes, both of which in the new species are relatively narrower and longer and with no (petiole) or reduced (postpetiole) lateral extensions. *Tetramorium nama***sp. nov.** is described here and a modification to the key presented by Mbanyana et al. (2018) to accommodate the additional species is provided. The potential threats to the species posed by alluvial diamond mining, livestock farming and climate change are discussed. A preliminary assessment of the conservation status of *Tetramorium nama***sp. nov** is presented and suggests that, dependant on determination of the range of the species, it is likely to be classified as Vulnerable (VU) or Endangered (EN) under IUCN Red List criterion B1ab(iii).

## Introduction

A field survey for an assessment of the potential impacts of proposed extended operating hours at the Oena Diamond Mine (ODM) in the Richtersveld National Park (RNP) on terrestrial invertebrate populations, was undertaken in September 2019. Sampling of ant populations by pitfall trapping and active searching in the ODM mineral lease area and nearby sites within the RNP yielded a total of 41 species. These included at least five undescribed species: one *Camponotus*, three *Monomorium* and one *Tetramorium*, the latter being the focus of this paper. The recognition of several potential threats to the survival of invertebrate species in the ODM mineral lease area and surrounding region leads to a need for formal conservation assessments which in turn leads to a requirement for formal descriptions of the new species.

The *T. solidum*-group, to which the new *Tetramorium* species belongs, was revised by Bolton (1980). He listed 13 species, later assigning *T. rothschildi* (Forel, 1907) as the 14^th^ species in the group upon the synonymy of *Triglyphothrix* under *Tetramorium* (Bolton 1985). The *T. solidum*-group was more recently revised by Mbanyana et al. (2018), who described five new species and presented an illustrated key to the 19 species then known. Another group-level revision in the near future is unlikely and description of a single additional species to enable identification and formal conservation assessment is thus justified. A description of *Tetramorium nama* sp. nov. and an additional couplet to be used in conjunction with the existing key in Mbanyana et al. (2018) to allow identification of the new species are therefore presented here. As this publication is intended to be used in conjunction with Mbanyana et al. (2018), the reader is referred to the latter for a full diagnosis and overview of the *T. solidum* species-group.

Members of the *T. solidum*-group are believed to be predominantly granivorous (Bolton 1980; Mbanyana et al. 2018), but no detailed studies on food preferences appear to have been done. The group is largely restricted to southern Africa and displays its greatest diversity in the arid south-western parts of this region, within which the RNP lies. Factors such as climate change, livestock grazing and habitat transformation by mining all have the potential to affect habitat and food availability within the RNP for species in this group. A preliminary assessment of the conservation status of *T. nama* sp. nov. and a discussion of potential threats to the survival of this and other ant species in the region are therefore also presented.

## Materials and methods

Pitfall trap sampling was carried out between 11 and 18 September 2019 in ten transects, each with 20 traps set at 10 m intervals and run for six days; the traps were each filled with 50 ml of a 1:1 mixture of 95% ethanol and propylene glycol. Transects were placed in each of the main habitat types (riverine fringe, alluvial terrace gravels, alluvial fans, aeolian sands, Mispah soils and mountain slopes) within the ODM mineral lease area, which extends for approximately 16 km along the banks of the Orange River on the northern border of the Richtersveld National Park. Specimens were collected under permit # RNP03/19 issued by SANParks. Representatives of each morphospecies recognised in the samples were subsequently point-mounted for identification.

Measurements of mounted specimens were taken using a Leica MZ16 stereomicroscope equipped with an axial shift carrier and an ocular graticule calibrated against a stage micrometer. Specimens were photographed using a Leica DFC 425 digital camera connected to a Leica Z16APO Macroscope. Images were captured using Leica Application Systems (LAS) multifocus V4.9; montage images were generated using Helicon Focus V6.8.0 and edited with Adobe Photoshop CS3.

### Terminology

Terminology relating to adult morphology largely follows Hita Garcia and Fischer (2014) as used by Mbanyana et al. (2018) with some differences as discussed below; descriptions of surface sculpture follow Harris (1979). All measurements (in millimetres) and indices are presented as minimum and maximum, with arithmetic means in parentheses. Abbreviations of the measurements taken and the indices based on them are as follows:

**Measurements**

**EL** Eye length: maximum diameter of the compound eye measured in oblique lateral view;

**HL** Head length: length of head measured in full-face view, from the midpoint of the clypeal margin to the midpoint of the occipital margin; where either of these margins is concave the measurement is taken from the midpoint of a line joining the anterior-most portions of the clypeus or the posterior-most portions of the occipital margin so that the impressions on these margins do not reduce HL;

**HL2** Head length 2: maximum distance from the midpoint of the anterior clypeal margin to the midpoint of the posterior margin of head, measured in full-face view; impressions on the anterior clypeal margin and the posterior head margin reduce HL2;

**HW** Head width: width of the head directly behind the eyes measured in full-face view;

**PH** Pronotal height: maximum height of the pronotum measured in lateral view;

**PPH** Postpetiole height: maximum height of the postpetiole measured in lateral view from the highest (median) point of the node to the ventral outline; the measuring line is placed at an orthogonal angle to the ventral outline of the node;

**PPL** Postpetiole length: maximum length of the postpetiole measured in lateral view;

**PPW** Postpetiole width: maximum width of the postpetiole measured in dorsal view;

**PSL** Propodeal spine length: in dorsofrontal view the tip of the measured spine, its base, and the centre of the propodeal concavity between the spines must all be in focus; using a dual-axis micrometer the spine length is measured from the tip of the spine to a virtual point at its base where the spine axis meets orthogonally with a line leading to the median point of the concavity;

**PTH** Petiolar node height: maximum height of the petiolar node measured in lateral view from the highest (median) point of the node to the ventral outline;

**PTL** Petiolar node length: maximum length of the dorsal face of the petiolar node from the anterodorsal to the posterodorsal angle, measured in lateral view;

**PTW** Petiolar node width: maximum width of the dorsal face of the petiolar node measured in dorsal view;

**PW** Pronotal width: maximum width of the pronotum measured in dorsal view;

**SL** Scape length: maximum scape length excluding basal condyle and neck;

**WL** Weber’s length: diagonal length of mesosoma in profile, from the junction of the pronotum and the cervical shield, to the posterior basal angle of the metapleuron.

**Indices**

**CI** Cephalic index: HW / HL × 100;

**CI2** Cephalic index 2: HW / HL2 × 100;

**DMI** Dorsal mesosoma index: PW / WL × 100;

**DPeI** Dorsal petiole index: PTW / PTL × 100;

**DPpI** Dorsal postpetiole index: PPW / PPL × 100;

**LMI** Lateral mesosoma index: PH / WL × 100;

**LPeI** Lateral petiole index: PTL / PTH × 100;

**LPpI** Lateral postpetiole index: PPL / PPH × 100;

**OI** Ocular index: EL / HW × 100;

**PeNI** Petiolar node index: PTW / PW × 100;

**PPI** Postpetiole index: PPW / PTW × 100;

**PpNI** Postpetiolar node index: PPW / PW × 100;

**PSLI** Propodeal spine index: PSL / HL × 100;

**PSLI2** Propodeal spine index: PSL / HL2 × 100;

**SI** Scape index: SL / HW × 100.

Note that 1) HL2 and the CI2 and PSLI2 indices derived from this are included for equivalence with HL, CI and PSLI as reported in Mbanyana et al. (2018), who in following Hita Garcia and Fischer (2014) used for HL the measurement here defined as HL2, 2) petiole node length and postpetiole length are more accurately measured in profile view than in dorsal view as described in Hita Garcia and Fischer (2014), and 3) PeNI and PpNI values as defined here and in Hita Garcia and Fischer (2014) were reported in Mbanyana et al. (2018) but the definitions were omitted. Measurements and indices of all specimens are available in Suppl. material. 1: File S1: Measurements_Tetramorium_nama.xls.

**Abbreviations of depositories**

**AFRC** AfriBugs Collection, Pretoria, South Africa

**BMNH** The Natural History Museum, London, UK

**CASC** California Academy of Sciences Collection, San Francisco, USA

**SAMC** Iziko South African Museum Collection, Cape Town, South Africa

### Additional couplet to insert in key of Mbanyana et al. (2018)

The thoroughly illustrated key provided in Mbanyana et al. (2018) contains 18 couplets to distinguish between 19 species; the couplets below are intended to be inserted at the position of couplet 8 in the existing key, with the additional couplet designated as 8a, to allow the remainder of the key to continue unaltered from couplet 9.

**Table:** 

8	Petiole node distinctly broader than long (DPeI 145–165)	**8a**
–	Petiole node usually broader than long, but not distinctly so, sometimes slightly longer than broad (DPeI 90–135)	**continue from couplet 9 in Mbanyana et al. (2018)**
8a	Mesosoma and petiole dorsally finely reticulate, postpetiole dorsum smooth to finely reticulate, with scattered piligerous foveolae, postpetiole narrower (DPpI 162–179)	***T. lerouxi* Mbanyana, Robertson & Hita Garcia, 2018**
–	Mesosoma dorsum longitudinally costulate, petiole dorsum with roughly concentric to irregularly transverse costulae, postpetiole transversely costulate, postpetiole broader (DPpI 184–201)	***T. nama* sp. nov.**

#### Tetramorium nama sp. nov.

Taxon classificationAnimaliaHymenopteraFormicidae

7EC81B13-AFBB-5E20-A962-E1C8CE2056F3

http://zoobank.org/95780860-8777-4385-BF0F-19FE0027A692

**Figure 1.**
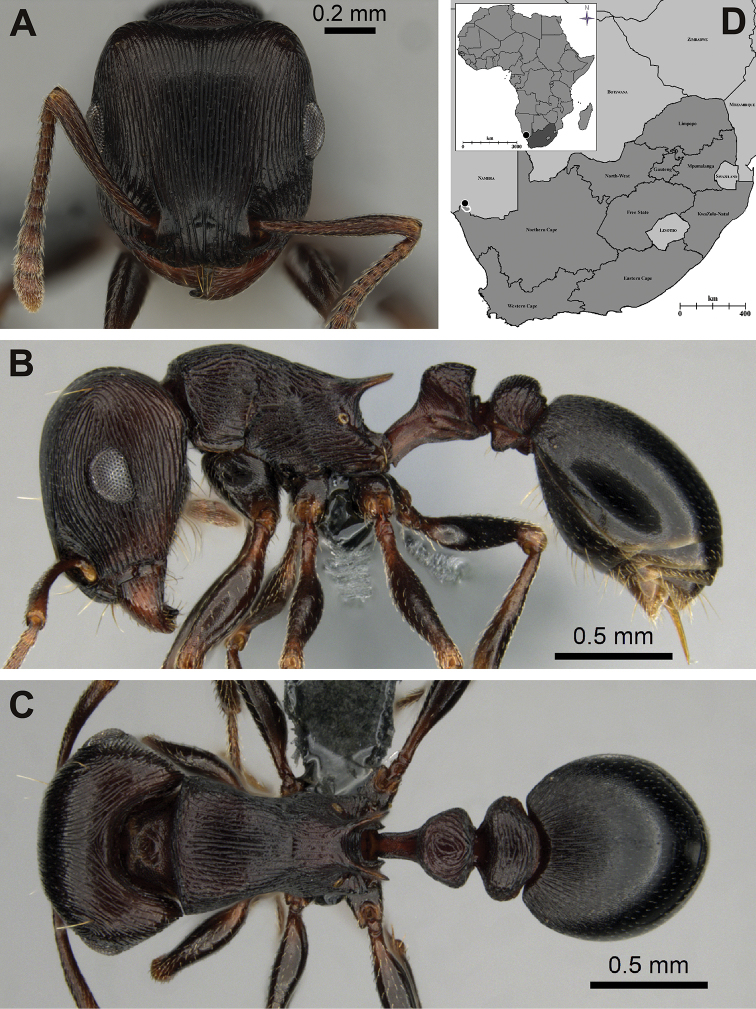
*Tetramorium nama* sp. nov. **A–C** holotype worker, CASENT0818990: **A** full-face view **B** lateral view **C** dorsal view **D** map of Africa (inset, with South Africa shaded dark grey) and detail of South Africa (main image), showing known distribution of *Tetramorium nama* sp. nov. (black dot), with a white outline indicating the Richtersveld National Park. (Photos by Bradley Reynolds, from www.AntWeb.org)

**Figure 2.**
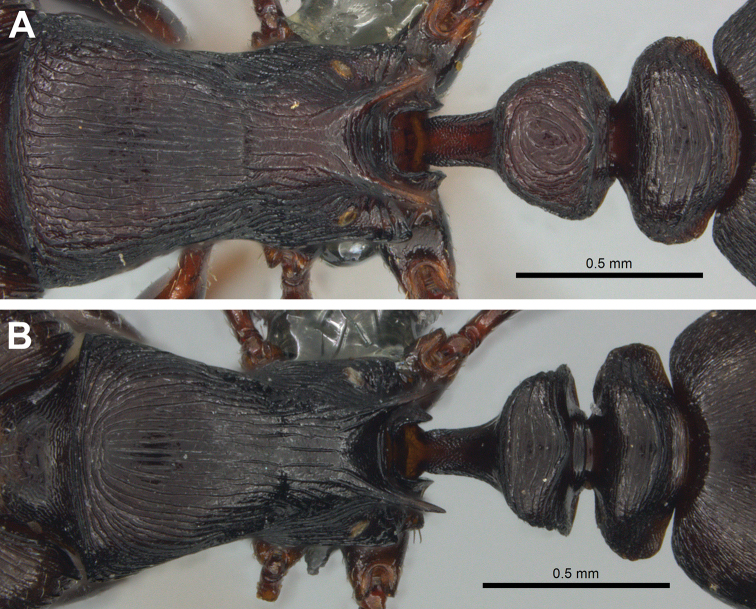
Dorsal view of mesosoma and petiole segments **A***Tetramorium nama* sp. nov. holotype worker, CASENT0818990 **B***T. grandinode*, CASENT0818846. (Photos by Peter Hawkes, from www.AntWeb.org)

**Figure 3.**
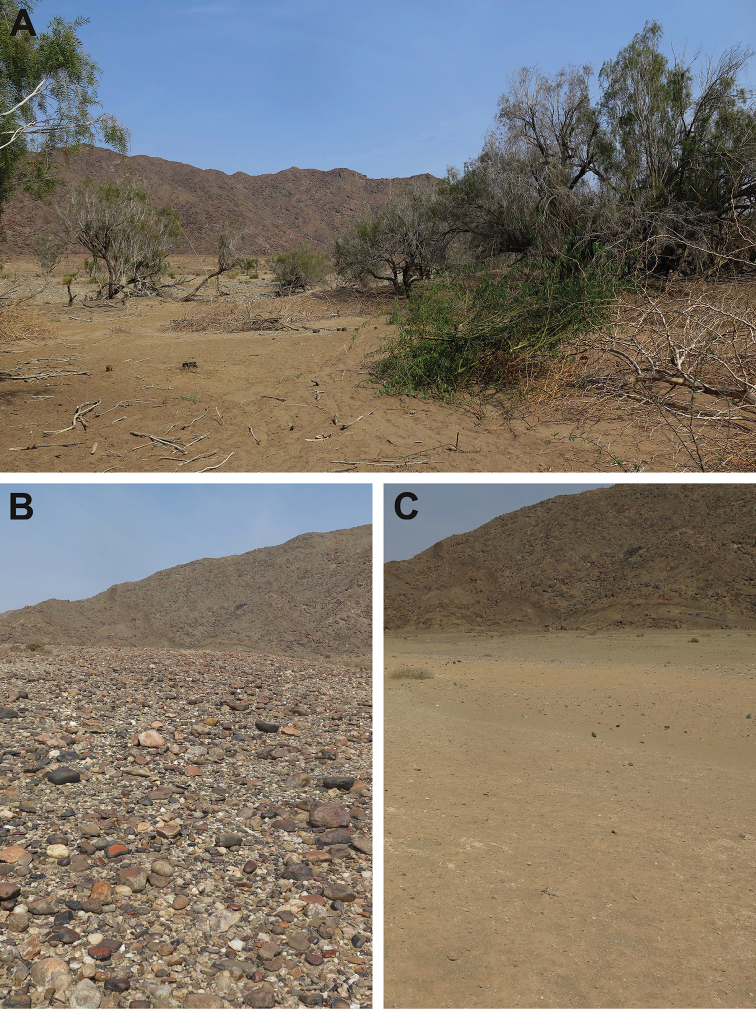
**A** riverine fringe habitat in which *Tetramorium nama* sp. nov. was collected, showing living vegetation dominated by *Euclea pseudebenus* as well as numerous cut *Prosopis glandulosa* (freshly cut and piles of dry branches) **B** alluvial terrace gravel and **C** alluvial fan habitats (in which *T. pogonion* and *T. rufescens* were collected) between the river and the rocky mountains to the southwest: the alluvial fan lies between the gravel terrace and the mountains.

Figures 1A–C , 2A , 3

##### Type material.

***Holotype*.** South Africa • **1worker**; Northern Cape Province, Namakwa, Richtersveld National Park; 28.05795S, 17.11256E ±100 m; alt. 65 ±10 m a.s.l.; P.G. Hawkes, D. Molenaar, R.N. Ungerer leg.; 12–18 Sep. 2019; Collection number: ODM2019-T8-1; Pitfall trap; Point-mounted; SAMC CASENT0818990.

***Paratypes*.** South Africa • **6 workers**; same data as for holotype; Point-mounted; AFRC CASENT0818735, CASENT0818736, BMNH CASENT0818992, CASC CASENT0818993, CASENT0818994, SAMC CASENT0818991; • **1 worker**; same data as for holotype; 95% ethanol; AFRC CASENT0819639.

##### Diagnosis.

**Workers.***Tetramorium nama* sp. nov. is morphologically most similar to *T. grandinode* Santschi, 1913, with which it shares almost identical cephalic sculpture, but can be easily separated from this species by the following characters: in *T. nama* sp. nov., the longitudinal costulae on the dorsum of the promesonotum run parallel and separate all the way to the anterior pronotal margin (Figure 2A), whereas in *T. grandinode* the costulae become strongly arcuate anteriorly, with lines from either side of the midline usually joining medially and forming a nested set of hyperbolic curves (Figure 2B); in *T. nama* sp. nov., the petiole dorsum is much longer and has irregular concentric or whorled to irregularly transverse costulate sculpture (Figure 2A), while in *T. grandinode* the petiole node is very short and has strong transverse costulae on its dorsal surface (Figure 2B). The petiole node in *T. nama* sp. nov. is narrower (only slightly wider than the distance between the propodeal spine tips) than in *T. grandinode* (in which the petiole node is ca. 1.4–1.5 times wider than the distance between the propodeal spine tips). From *T. lerouxi* Mbanyana, Robertson & Hita Garcia, 2018, the only other glabrous species with the petiole node distinctly laterally expanded, *T. nama* sp. nov. can be readily distinguished by the sculpture of the dorsal mesosoma and petiole segments as indicated in the key. *T. nama* sp. nov. shares similar head, mesosoma, petiole and postpetiole sculpture with *T. duncani* Mbanyana, Robertson & Hita Garcia, 2018, but can be distinguished from this species as the former has substantially longer propodeal spines (PSLI2 23–25 vs. 12–17) a much shorter petiole node (LPeI 66–70 vs. 78–96), a distinct subpetiolar process (which *T. duncani* lacks) and a much less pronounced sub-postpetiolar process (very well-developed in *T. duncani*).

It should be noted that some specimens referred by Mbanyana et al. (2018) to *T. grandinode* have substantially narrower petiole and postpetiole nodes than seen in typical *T. grandinode* sensu Bolton (1980). As a result, Mbanyana et al. (2018) reported for *T. grandinode* extreme ranges of PeNI (56–83) and PpNI (68–110) which respectively overlap with the ranges they presented for 13 and eight of the 18 other *solidum*-group species they listed, as well as those of *T. nama*. However, Mbanyana et al. (2018) did not formally integrate their atypical material into *T. grandinode* and provide an updated and expanded description to accommodate these specimens. Both their key and diagnosis thus follow Bolton (1980) in emphasising the extremely broad petiole and postpetiole nodes as the main identifying characters for this species. Any atypical specimens listed by Mbanyana et al. (2018) with PpNI near or below that of *T. lerouxi* (PpNI 78–80) or *T. nama* sp. nov. (PpNI 71–76) will thus fail to key to *T. grandinode* and cannot be considered to fall within this species as defined by the most recent description (Bolton 1980) and diagnosis (Mbanyana et al. 2018). *Tetramorium nama* sp. nov. is therefore distinguished here from typical *T. grandinode* only. The placement of the atypical material referred to the latter by Mbanyana et al. (2018) requires future re-evaluation; some of this material may prove to belong to *T. nama* sp. nov, and/or may represent additional undescribed species.

##### Description.

**Workers. Measurements. *Holotype*** CASENT0818990: HL 1.21, HL2 1.17, HW 1.10, SL 0.85, EL 0.31, PH 0.56, PW 0.74, WL 1.32, PSL 0.28, PTH 0.42, PTL 0.29, PTW 0.43, PPH 0.42, PPL 0.29, PPW 0.56. **Indices**: CI 91, CI2 94, DMI 56, DPeI 147, DPpI 191, OI 28, SI 77, LMI 42, PSLI 23, PSLI2 24, PeNI 58, LPeI 70), LPpI 69, PpNI 76, PPI 130 (all measurements in mm, indices as percentage).

***Paratypes*** (6 measured, mean including holotype in parentheses): HL 1.14–1.22 (1.19), HL2 1.11–1.19 (1.15), HW 1.04–1.13 (1.09), SL 0.80–0.86 (0.83), EL 0.30–0.33 (0.32), PH 0.54–0.56 (0.55), PW 0.68–0.75 (0.73), WL 1.17–1.32 (1.24), PSL 0.26–0.29 (0.27), PTH 0.38–0.43 (0.41), PTL 0.27–0.29 (0.28), PTW 0.39–0.44 (0.42), PPH 0.39–0.43 (0.42), PPL 0.26–0.29 (0.28), PPW 0.49–0.56 (0.53). **Indices**: CI 90–93 (92), CI2 94–96 (95), DMI 58–60 (59), DPeI 148–158 (152), DPpI 184–201 (194), OI 28–30 (29), SI 74–77 (76), LMI 44–46 (44), PSLI 22–24 (23), PSLI2 23–25 (24), PeNI 56–60 (58), LPeI 66–70 (68), LPpI 64–69 (66), PpNI 71–75 (73), PPI 124–127 (126) (all measurements in mm, indices as percentage).

***Head*** a little longer than wide (CI 92), posterior margin shallowly indented medially, sides slightly convex, rounding posteriorly into the broadly convex occipital corners. Clypeus with a distinct but shallow medial indentation and ca. 10–12 longitudinal costulae overlain on a very effaced shagreenate ground sculpture, the surface between the costulae almost smooth, moderately shining. Frontal carinae fading out behind the frontal lobes and merging with the remaining cephalic sculpture from the level of the anterior margin of the eyes, often broken behind this point, but occasionally fairly long and reaching to midway between the level of the posterior margin of the eyes and the occipital margin. Eyes large, situated at approximately the mid-length of the sides of the head and with 16–17 ommatidia in the longest row, ocelli absent. Mandibles weakly longitudinally rugose, with seven teeth. Psammophore well-developed, comprising a row of ca. 10–14 elongate J-shaped hairs on the ventral surface of the head behind the posterior margin of the buccal cavity, two rows of ca. five hairs each along the distal inner and outer ventral margins of each mandible and a cluster of four or five hairs proximally on the ventral mandibular margins. Antennal scapes short, stout, basally curved and distally thickened; when laid back, scapes fall short of the posterior margin of the head by ca. one-fifth of their length. Scapes with strong appressed pubescence, lacking erect setae, remaining antennal segments with sub-appressed pubescence. Scapes weakly longitudinally carinulate, the remaining antennal segments smooth and shining, unsculptured except for piligerous punctures. Dorsal surface of head with dense longitudinal costulae, ca. 22 between the frontal carinae at the level of the mid-point of the eyes; costulae approximately parallel medially but diverging posterolaterally toward the occipital corners, behind which they reflex and continue anterad on the lateral surface of the head, a few above, but most below the eyes. Spaces between the costulae shiny and almost smooth, with faint reticulate/shagreenate sculpture.

***Mesosoma*** laterally with irregular longitudinal costulae overlaid on a weak reticulate-punctate ground sculpture, anterodorsally with one continuous and several broken transverse marginal costulae; dorsally with effaced reticulate-punctate ground sculpture overlain by fairly uniform longitudinal costulae that continue running approximately parallel all the way to the pronotal margin, where they either fade out or join approximately at right angles with one of the transverse components. Propodeal spiracles round, situated slightly above the mid-height of the sides of the propodeum. Propodeal spines long and acute (PSLI 22–24). Declivity dorsally shallowly concave in dorsal view but straight ventrally. Ground sculpture of propodeum shagreenate to reticulate-punctate, overlain by costulae which are longitudinal on the dorsal face but transverse on the declivitous face and between the bases of the propodeal spines. Propodeal lobes broadly rounded.

***Petiole*** node in dorsal view subtrapezoidal, distinctly wider behind than in front and distinctly wider than long, in profile with a broadly rounded anterodorsal margin and an acute, slightly overhanging posterodorsal margin. Subpetiolar process a narrow lamella subtended by a short but distinct anteriorly orientated tooth. Dorsal surface of node usually with several irregular roughly concentric or whorled costulae, in some specimens the costulae are irregularly transverse.

***Postpetiole*** node distinctly broader than the petiole node in dorsal view, with irregular, slightly recurved transverse costulae over the entire dorsal surface, the anterior face of the node slightly concave, the posterior face slightly convex.

***Gaster*** with dense but fine basigastral costulae radiating over the anterior third of the first tergite, overlain on a dense shagreenate ground sculpture which weakens posteriorly. Sting present, weakly curved ventrad, with a distinct pennant-shaped lamellate appendage.

***Legs*** with fairly uniform shagreenate sculpture throughout.

***Pilosity***: two pairs of erect setae are present on the dorsal surface of the head behind the frontal lobes; one about level with the anterior margin of the eyes and one close to the occipital corners. Standing hairs are absent from all dorsal surfaces of the mesosoma, petiole segments, the first and usually the second gastral segment (the latter occasionally with one to two pairs of very short suberect setae). Dorsal surfaces of head, petiole, postpetiole and gaster with scattered appressed pubescence, which is very sparse, reduced in length and appearing virtually absent from the dorsal mesosoma. Femora and tibiae with appressed to sub-appressed pubescence on all surfaces. Gastral sternites with appressed pubescence and long erect setae, which on the first sternite are concentrated medially and posteriorly.

***Colour*** mainly dark blackish brown, the mandibles a distinctly contrasting reddish brown; antennae, legs and peduncle of petiole similar to the mandibles but with the median portions of the femora and tibiae darker and similar to the body colour.

The type series is very consistent in overall appearance and colour, but there is some variation in the pattern of sculpture on the petiole node as indicated above. Worker measurements and proportions are quite consistent with measurement ranges 4–13% and index ranges 2–9% of their mean values.

**Queen and male unknown.**

##### Etymology.

*nama* refers to the local Nama people who, together with SANParks, jointly manage the Richtersveld National Park. The specific epithet is a noun in apposition and is thus invariant.

##### Habitat and distribution.

*Tetramorium nama* sp. nov. is known only from a single collection of eight workers from one pitfall trap, which was situated in an area transitional between Lower Gariep Alluvial Vegetation and Noms Mountain Desert (Mucina and Rutherford 2006). The pitfall trap transect was located in open vegetation between closed thicket on the banks of the Orange River and alluvial terrace gravels further from the river. The site is located in the extreme north of the RNP in the Namakwa district of the Northern Cape, South Africa; more extensive surveys in the surrounding region are required to determine the extent of occurrence of the species.

## Discussion

### Related species, habitat and distribution

Specimens representing three other species within the *Tetramorium solidum*-group were also found during the 2019 survey of the Oena Diamond Mine; these included three specimens of *T. grandinode* Santschi, 1913, 113 of *T. pogonion* Bolton, 1980 and 390 of *T. rufescens* Stitz, 1923. *Tetramorium grandinode* and *T. rufescens* are fairly widespread and are known respectively from at least 22 and 38 other sites in Namibia and South Africa (Bolton 1980; Mbanyana et al. 2018; AntWeb 2020), but the *T. pogonion* specimens represent only the second record of this species and the first record in South Africa. *Tetramorium nama* sp. nov. and *T. grandinode* were rare in the ODM project area and found in only one and two of the 200 pitfall trap samples respectively, while *T. pogonion* and *T. rufescens* were relatively common and were found in 22 and 40 samples respectively. The habitat in which the *T. nama* sp. nov. and *T. grandinode* specimens were found was open riverine fringe vegetation dominated by two indigenous tree species, *Euclea pseudebenus* E. Mey. ex A. DC (Black Ebony) and *Tamarix usneoides* E. Mey. ex Bunge (Wild Tamarisk), although a number of other tree species were present in smaller numbers. Substantial invasion by *Prosopis glandulosa* Torr. (Honey Mesquite) has occurred in the area and is the subject of an intensive eradication campaign by the Department of Environmental Affairs’ Working for Water (WfW) programme; many cut and poisoned stumps, as well as piles of both dry and freshly cut growth were observed within the survey area (see Figure 3).

*Tetramorium nama* sp. nov. was found only within the riverine fringe vegetation during the September 2019 ODM survey, but the single record provides insufficient information to indicate whether or not the species might inhabit other areas within the RNP. Two other seed-harvesting species, *T. pogonion* and *T. rufescens*, each occurred in five of the six habitat types sampled, together inhabiting all habitat types and suggesting the potential suitability of these habitats for *T. nama* sp. nov. as well. The absence of any live grasses and apparent dormancy of all non-tree species in Figure 3 highlights the importance of seed storage by *T. solidum*-group species in ensuring their survival through drought periods. Further, more intensive, surveys would be required to detect habitat preferences of *T. nama* sp. nov.

The distribution of *T. nama* sp. nov. beyond the type locality also cannot be determined at this stage, but this species was absent from all of the institutional collections mentioned in Mbanyana et al. (2018) and was not found during any of the targeted surveys that these authors carried out in the Northern Cape and Namibia for *T. solidum*-group species. No specimens of this species were found during a survey of 48 sites in the Central Namib Desert in 2010 (circa 500 km north of the type locality), nor in several intensive surveys on and around Gamsberg (circa 200 km SE of the type locality) in the Bushmanland Inselberg Region of the Northern Cape from 2009–2017 (unpublished data, AFRC). This suggests that *T. nama* sp. nov. has a limited distribution, or is very rare within its range, or possibly both. It is however fairly certain that the species will occur in southern Namibia, even if only in habitat similar to where it was found, on the opposite banks of the Orange River.

### Conservation and threats

**The future of the Richtersveld National Park**

The RNP is situated in the extreme northern part of the Northern Cape Province in South Africa and forms part of the |Ai-|Ais/Richtersveld Transfrontier Park. The RNP is unusual in that human inhabitants and alluvial diamond mining operations have continued to occupy the park since its proclamation in 1991. The South African National Parks (SANParks) manages the park as a communal pasture on a contractual basis, and 26 registered farmers from the local Nama community have the right to graze the equivalent of a total of 6,600 head of small livestock units (SLU) within the park boundaries.

The initial 30-year contract park agreement that came into effect in 1991 will expire in 2021, but a new agreement has been negotiated and is expected to be signed in the near future, thereby ensuring continued protection of the park for at least another 30 years. The extreme aridity of the environment means that alternative uses are unlikely to be viable and the park is thus likely to persist in the long-term.

### Climate change

The climate of the RNP is harsh; mean annual precipitation (MAP) ranges from ca. 55 mm in the north and northeast to ca. 125 mm in the southwest of the park (WorldClim dataset, Hijmans et al. 2005), with peak temperatures at times exceeding 50 °C (SANParks 2018). In addition, rainfall is highly sporadic and patchy and some localities may receive no rain for several years; a site in the Tatasberg had no rainfall recorded from 2000–2016, during which period the average MAP for the park interior was 38 mm and one site received 163 mm MAP (SANParks 2018). While species endemic to the area clearly must be adapted to such conditions, changing climate may result in some species’ tolerances being exceeded.

From 1990 to 2010 the RNP had already experienced an increase of 1.2 °C in mean maximum and 1.1 °C in mean minimum temperatures, exceeding the near-future (2035) predictions utilised by the South Africa Department of Environmental Affairs (DEA) for planning purposes, and there had been a significant increase in the number of days exceeding 35 °C (Wilgen et al. 2015). In conjunction with no change in MAP (but with a higher number of smaller rainfall events) this indicates an increase in aridity in the region from 1990–2010. A subsequent severe drought from 2016 to present, during which Sendelingsdrift (23 km west of the type locality of *T. nama* sp. nov.) has received an annual average of just over 6% of its historic MAP for four years, has resulted in large-scale vegetation loss. Even extreme arid-adapted species such as the three quiver tree species (*Aloidendron dichotomum* (Masson) Klopper & Gideon F. Sm., *A. pillansii* (L. Guthrie) Klopper & Gideon F. Sm. and *A. ramosissimum* (Pillans) Klopper & Gideon F. Sm.) present in the park have experienced extreme die-off, with an approximately 70% reduction in numbers of live trees (B. Whittington, pers. comm.). If such changes continue and the further temperature rises predicted for the coming decades occur, the RNP will become increasingly desert-like (SANParks 2018).

It is thus likely that local conditions could become unsuitable for species such as *Tetramorium nama* sp. nov. and they would have to track suitable habitat and climatic conditions in order to survive. Conditions suitable for nuptial flights often would not occur for periods of several to many years in the northern RNP and it is only by flighted dispersal that these ants could migrate far enough to track substantial climatic changes. Given the rapidity of recent climatic change it is possible that they might not be able to respond quickly enough and might therefore become locally or even globally extinct.

### Mining

Alluvial diamond mining has been carried out along the banks of the Orange River in the Richtersveld since around 1900; mining at Oena started in 1992, though the mine has changed ownership since then and is currently operated by African Star Minerals (ASM). Mining operations have been carried out at three areas (Oena, Sandberg and Blokwerf) within the mineral lease area, but have at least temporarily been suspended at one (Blokwerf). Mining operations are opencast, with topsoil (where present) being stripped and stockpiled, after which the alluvial gravels are excavated, screened and processed through rotary pans to provide concentrate for extraction in the recovery plant. Approximately 80% of the coarse waste is returned to the excavated areas to be used as backfill, with the remainder deposited in waste rock dumps. The fine tailings slurry is deposited in a dam and will eventually either be covered with coarse tailings or returned to excavated areas as backfill. The backfilled pits are covered with topsoil where this is available (topsoil is absent from much of the terrace gravel areas) and the area re-contoured to a natural-looking state. However, virtually all plants and animals within the mined deposits and in areas where the processed gravels are dumped will be killed. The resulting soil structure will also probably differ very substantially from the natural alluvial deposits and rehabilitation can be expected to be extremely slow given the arid nature of the environment. All mined areas and those covered by waste rock or tailings are therefore effectively lost as habitat for indigenous species for a considerable period, which may be many decades. The area likely to be disturbed by currently planned mining activities constitutes only ca. 20% of the alluvial plains within the Oena mineral lease area, but this could be expanded (prospecting is continuing at two sections upstream of the three areas listed above) and a significant area had been disturbed by previous operations and has not yet been rehabilitated; this is to be rectified as part of the current environmental management plan (ASM 2018). The total area of habitat loss could therefore be a significant proportion of the total available.

The potential impact of artificial lighting of mining operations on *Tetramorium nama* sp. nov. cannot be determined at this stage as it is not known whether the nuptial flights of this species take place during the day or night. Impacts on nocturnal nuptial swarms could be severe, especially considering the extremely sporadic nature of suitable conditions for such events. Dust is another potentially significant factor that may reduce food availability via impacts on plants.

### Livestock grazing

Members of the local Nama community run herds primarily of domestic goats, *Capra aegagrus hircus* (Linnaeus, 1758), with the total domesticated livestock population within the RNP capped at 6,600 small livestock units (a stocking rate of approximately 25 ha per SLU). However, the riverine fringe along the banks of the Orange River where *Tetramorium nama* sp. nov. was collected is probably the most intensively utilised habitat within the park, as it provides fodder during the long dry periods when the vegetation further from the river provides very little forage (SANParks 2018).

Degradation of vegetation by goat overgrazing is recognised to be more severe than that from any other ruminant livestock species, due to their ability to graze on residual biomass and woody species that would be left as vegetation cover by other livestock (Steinveld et al. 2006), as well as their ability to debark trees, range over large distances and survive without water for longer periods than other livestock (Lipson et al. 2011).

Intensive grazing even by domesticated goats would be expected to substantially reduce seed set by grasses and shrubs that provide food for *Tetramorium solidum*-group species. Continuation of the current trend of climate change is likely to reduce carrying capacity in the RNP and lead to an increased risk of overgrazing; regular review of grazing quotas should ideally be carried out as part of the park’s Environmental Management Plan. Should feral populations of goats become established in the park, the cap on numbers would become very difficult to maintain and impacts could become far more severe, potentially virtually eliminating the food supply of granivorous ants and putting them at high risk of at least local extinction.

However, *T. signatum* Emery, 1895, another *T. solidum*-group species, has been observed (unpublished data) preying on live *Microhodotermes viator* (Latreille, 1804), which suggests that members of the group might not be wholly dependent on seeds as a food source. The extent to which termites and perhaps other insect prey can substitute for their normal food is unknown, but this may at least partially mitigate the impact of livestock such as goats on their food supply; further research is required.

### Conservation assessment

Distribution data are, at present, too limited to allow a full IUCN Red List assessment (IUCN 2012) to be carried out and *T. nama* sp. nov. should currently be considered Data Deficient (DD). However, it is very likely that the species will prove to be restricted to the northernmost parts of the Northern Cape and southernmost parts of Namibia, given the lack of any records of the species from numerous sampling events to the north and south of this region. In light of the multiple threats discussed above (each of which is expected to contribute to continuing decline in quality and extent of habitat), even if the range proves to be as much as four times that of the entire RNP, the Extent of Occurrence (EOO) would fall within the range (< 20,000 km^2^) for the species to be classified as Vulnerable (VU) under criterion B1ab(iii). The species may however be limited to a narrower region straddling the Orange River, which would lead to an Endangered (EN) classification under the same criterion if the EOO proves to be less than 5,000 km^2^. Other ant species might be similarly threatened by the factors discussed here, although the impact of grazing would be expected to vary depending on their reliance on seeds or other plant-related food sources (such as honeydew from phytophagous homopterans).

## Conclusions

The discovery of an undescribed *Tetramorium solidum*-group species so soon after the publication of a revision of the group, for which a substantial number of targeted surveys in the region had been carried out, suggests that there may be more short-range endemic species within the group inhabiting the ecologically complex Richtersveld region.

*Tetramorium nama* sp. nov. is likely to prove to be both rare and limited in range; together with the threats posed by climate change and livestock grazing, as well as current and planned mining activities in the |Ai|Ais/Richtersveld Transfrontier Park, it is thus likely that a formal assessment would indicate a categorisation of VU or higher following the IUCN criteria. Formal Red List assessments of the conservation status of range-restricted species such as *T. nama* sp. nov. may assist in enabling action to be taken to protect sufficient areas of natural habitat to ensure their continued survival and such assessments should be undertaken as a matter of urgency.

## Supplementary Material

XML Treatment for Tetramorium nama

## Supplementary materials

Supplementary material 1 File S1 Data type measurement https://binary.pensoft.net/file/449152This dataset is made available under the Open Database License (http://opendatacommons.org/licenses/odbl/1.0/). The Open Database License (ODbL) is a license agreement intended to allow users to freely share, modify, and use this Dataset while maintaining this same freedom for others, provided that the original source and author(s) are credited.Peter G. Hawkes
